# Enhancing power quality of pv grid-connected system through mantis shrimp optimization algorithm for optimal Dc bus voltage control

**DOI:** 10.1038/s41598-025-32058-y

**Published:** 2026-01-19

**Authors:** Alla Eddine Boukhdenna, Hamza Afghoul, Djallal Eddine Zabia, Feriel Abdelmalek, Yakoub Nettari, Salah S. Alharbi, Saleh S. Alharbi

**Affiliations:** 1 Laboratory of Technologies of Energetic Systems E3360100, Department of EEA, National Higher School of Technology and Engineering, Annaba, Algeria; 2 LAS laboratory, Automation and intelligent systems department, Faculty of Technology, University of Setif 1- Ferhat ABBAS, Setif, Algeria; 3 LI3CUB Laboratory, University of Biskra, Biskra, Algeria; 4 LEPCI laboratory, Department of Electronics, Setif 1 University- Ferhat ABBAS, Setif, Algeria; 5 Department of Electrical and Electronics Engineering, Istanbul Sabahattin Zaim University, Istanbul, Turkey; 6 Department of Electrical Engineering, Faculty of Engineering, Al-Baha University, Alaqiq, 65779 Saudi Arabia

**Keywords:** DC bus reference voltage, Predictive direct power control, Photovoltaic systems, Mantis shrimp optimization algorithm, Shunt active power filter, Total harmonic distortion, Energy science and technology, Engineering

## Abstract

The nonlinear and intermittent nature of Photovoltaic (PV) systems introduces dynamic disturbances that negatively impact the stability of the DC bus voltage (*V*_*dc*_) between PV sources and shunt active power filters (SAPFs). These fluctuations pose significant challenges to the performance of SAPFs, especially when the reference DC bus voltage (*V*_dc_*) is constant and not adapted to the instantaneous operating conditions. In this study, a Perturb and Observe (P&O) algorithm is employed within the PV subsystem to perform Maximum Power Point Tracking (MPPT), further contributing to the time-varying behavior of *V*_*dc*_. To address this problem, this paper proposes a real-time optimization strategy based on the Mantis Shrimp Optimization Algorithm (MShOA) for continuous *V*_dc_* adjustment. This method relies on real-time Total Harmonic Distortion (THD) feedback to dynamically determine the optimal *V*_dc_*, thereby improving harmonic mitigation and maintaining voltage stability. Simulation results demonstrate that the proposed MShOA-based approach effectively reduces THD from 3.59% to 2.85% obtained with conventional methods to 2.33% before PV injection, and maintains 4.19% after PV injection, remaining within the IEEE 519 − 92 standard limits. To confirm its superiority, a comparison with the Whale Optimization Algorithm (WOA) was performed, which achieved 2.65% before and 5.78% after PV injection. These findings validate the higher accuracy, faster convergence, and better adaptability of the proposed MShOA in ensuring robust voltage regulation and improved power quality under PV injection conditions.

## Introduction

The integration of renewable energy sources, particularly photovoltaic (PV) power systems, into the electrical grid has led to a noticeable increase in harmonics, undesirable distortions in voltage and current waveforms, and a decrease in power factor, which weakens power quality. Due to the intermittent nature of solar irradiation, the energy input from PV sources to the grid can fluctuate significantly over short time periods, which may cause difficulties in maintaining grid stability^1^. Moreover, in PV systems, environmental variations such as partial shading can create multiple local maxima in the power-voltage curve, complicating the tracking of the global maximum power point. Metaheuristic optimization algorithms, such as Particle Swarm Optimization (PSO) and Whale Optimization Algorithm (WOA), have shown strong capability in overcoming such nonlinear challenges, further demonstrating their relevance for improving control performance in PV systems. To address these negative phenomena associated with PV power injection, especially in systems that contain nonlinear loads, the Shunt Active Power Filter (SAPF) emerges as an effective solution for improving power quality by injecting compensating currents into the grid^2^.

Control strategies significantly influence the performance of the SAPF. The Predictive Direct Power Control (PDPC) strategy is often used due to its structural simplicity and fast response^3^. This strategy relies on calculating the instantaneous flow of active and reactive power to control the compensating currents, without the need for explicit current control loops or Pulse Width Modulation (PWM) blocks, thereby maintaining compensation quality^4^. The choice of DC bus reference voltage (*V*_dc_*) greatly affects the effectiveness and stability of the active power filter, especially under variable load conditions and power disturbances. The appropriate selection of the *V*_dc_* ensures sufficient voltage headroom for the Voltage Source Inverter (VSI) to generate the necessary compensating current waveforms that oppose the harmonic currents and reactive power components generated by nonlinear loads^5^. If the value of *V*_dc_* is set lower than what is suitable for the system, the inverter cannot produce the required output current, particularly in high harmonic content conditions, leading to degradation in filtering performance and an increase in Total Harmonic Distortion (THD). In extreme cases, the inverter may completely lose its ability to inject compensating currents. On the other hand, a significant increase in the value of *V*_dc_* raises the voltage stress on power electronic components such as IGBT or MOSFET transistors, increasing the likelihood of thermal failures and reducing the overall reliability of the system. Moreover, high DC voltages lead to increased switching losses and Electromagnetic Interference (EMI), which reduces the energy efficiency of the SAPF and necessitates stricter solutions for thermal management. Therefore, the value of *V*_dc_* must be considered carefully to achieve a balance between providing sufficient dynamic range for harmonic compensation and minimizing energy losses and stress on power electronic components^6^.

### Literature review and motivation

The DC bus voltage (*V*_*dc*_) is regulated using a PI controller. However, the efficiency of the controller is highly sensitive to the reference voltage value *V*_dc_*. The injection of PV power into the grid causes dynamic disturbances, these disturbances directly affect the DC link voltage and may cause instability in its regulation if *V*_dc_* is not properly adjusted in real time. This leads to an increase in harmonic content and a reduction in the overall power factor. To address these issues. The research^7^ proposed an Equilibrium Optimized (EO) PI controller to regulate the *V*_*dc*_ in grid connected PV systems. The EO algorithm was employed to minimize *V*_*dc*_ variations during dynamic conditions, such as irregular PV power generation caused by insolation fluctuations. While the technique showed improved THD and power factor compared to traditional methods, its controller tuning was performed offline, limiting adaptability to real-time changes. Additionally, this study focused mainly on improving the performance of the controller itself, ignoring the impact of the *V*_dc_* on the overall system performance, resulting in reduced voltage error and improved power quality under dynamic disturbances. In^8^, a robust control strategy was proposed to regulate the *V*_*dc*_ of shunt active power filters without requiring load current measurements. The method, based on adaptive pole placement combined with a variable structure control scheme. However, the approach assumes a fixed *V*_dc_* and does not address the sensitivity of system performance to its value. This limitation may lead to suboptimal harmonic compensation and power quality degradation under varying operating conditions, particularly in systems with high PV penetration, where real-time adaptation of *V*_dc_* becomes essential. In contrast, the research^9^ introduced a theoretical approach to determine the appropriate value of *V*_dc_*, relying on the system’s load capacity and the highest-order harmonic to be compensated. Although systematic, this method neglected essential factors such as the internal resistance of the SAPF, which affected the precision of the calculated *V*_dc_*. Moreover, some techniques rely on parameters such as the Maximum Filter Terminal Voltage $({V_f}_{ - \max })$ that are typically obtained through simulations, making them highly sensitive to environmental changes and system reconfigurations. Such dependencies can result in inaccurate estimation of the *V*_dc_*, ultimately impairing the effectiveness of harmonic compensation and degrading overall power quality.

Despite these contributions, several critical research gaps remain unaddressed and are the focus of the present study:

- Poor performance under dynamic conditions: Most existing analytical approaches for determining *V*_dc_* are designed with fixed parameters and do not adapt in real time to variations caused by nonlinear loads or renewable energy injections.
- Neglect of *V*_dc_* optimization in controller design: While previous research has focused on adjusting the gains of PI controllers using intelligent algorithms, the optimal selection of *V*_dc_* itself remains insufficiently explored.
- Insufficient consideration of real-time power quality indicators: Previous strategies often fail to incorporate real-time indicators, such as THD, as part of the control strategy to determine *V*_dc_*. limiting the system’s responsiveness to actual power quality conditions.
- Limited validation in realistic disturbance scenarios: Many studies validate their approaches only under steady-state conditions, without taking into account the grid disturbances common in PV integrated environments.

In response to these identified gaps, the present work proposes a real-time *V*_dc_* optimization strategy by leveraging the Mantis Shrimp Optimization Algorithm (MShOA), aiming to enhance the harmonic mitigation and power quality performance of SAPF.

### Contributions and framework

Considering the abovementioned challenges in view, particularly those arising from the dynamic behavior of PV power injection, this research makes the following key contributions:

1. Development of a novel real-time optimization strategy for dynamically adjusting the *V*_dc_* in a three-phase SAPF, using the MShOA. This strategy includes an online adaptive mechanism that continuously selects the optimal *V*_dc_* based on real-time THD feedback.
2. A comparative evaluation with existing theoretical approaches for determining *V*_dc_*, highlighting the superior performance of the proposed strategy in reducing THD and maintaining power quality.
3. An additional evaluation of control efficiency, in which the proposed method is benchmarked against the WOA, highlighting enhanced convergence behavior and adaptability.
4. Comprehensive simulation-based validation, demonstrating the robustness of the proposed method under PV conditions.

The remaining sections of this paper are organized as follows: Section II presents the overall system configuration and control structure of the SAPF. Section III discusses the influence of PV power injection on the stability of the *V*_*dc*_. Section IV introduces the proposed approach for selecting the optimal *V*_dc_* using the MShOA. Section V illustrates the simulation results obtained under various operating scenarios. Section VI provides a comparative evaluation between traditional methods and the proposed technique. Finally, Section VII concludes the paper and outlines future research directions.

### System description

**Fig. 1:**
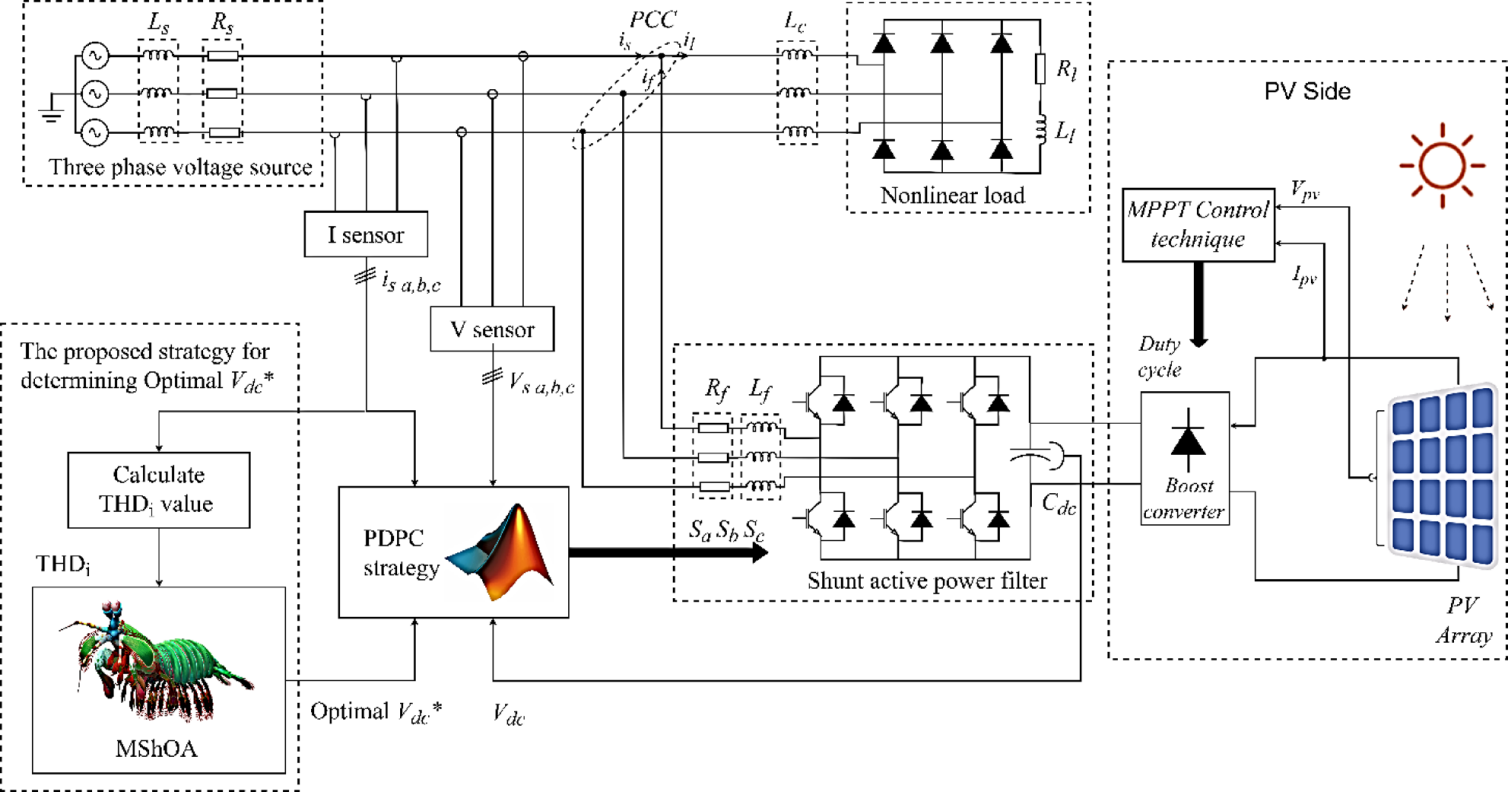
Configuration of the studied system.

The system integrates PV power with the electrical grid, as shown in Fig. 1, focusing on improving power quality and reducing harmonic distortions caused by nonlinear loads. This is achieved using SAPF. The harmonic components present in the load current ${i_l}\left( t \right)$, as shown in Eq. (1), significantly contribute to the THD, which can be measured using Eq. (2). The SAPF generates a compensating current ${i_f}\left( t \right)$aimed at reducing these harmonic components, ensuring that the source current ${i_s}\left( t \right)$remains as close as possible to a pure sinusoidal waveform^10^.

1 $${i_l}\left( t \right)={i_1}\sin \left( {\omega t+{\varphi _1}} \right)+\sum\limits_{{n=2}}^{\infty } {{i_n}} \sin \left( {n\omega t+{\varphi _n}} \right)$$

Where:

${i_1}$ Fundamental current at frequency ,

${i_n}$ Current harmonic at frequency ,

${\varphi _n}$ Phase shift of each harmonic component.

2 $$THD=\frac{{\sqrt {\sum\nolimits_{{n=2}}^{\infty } {I_{n}^{2}} } }}{{{I_1}}} \times 100\%$$

The control technique is a fundamental element in enhancing the performance of the SAPF, as it is carefully selected to ensure the highest levels of efficiency in improving power quality^11^.

### PDPC control strategy

**Fig. 2:**
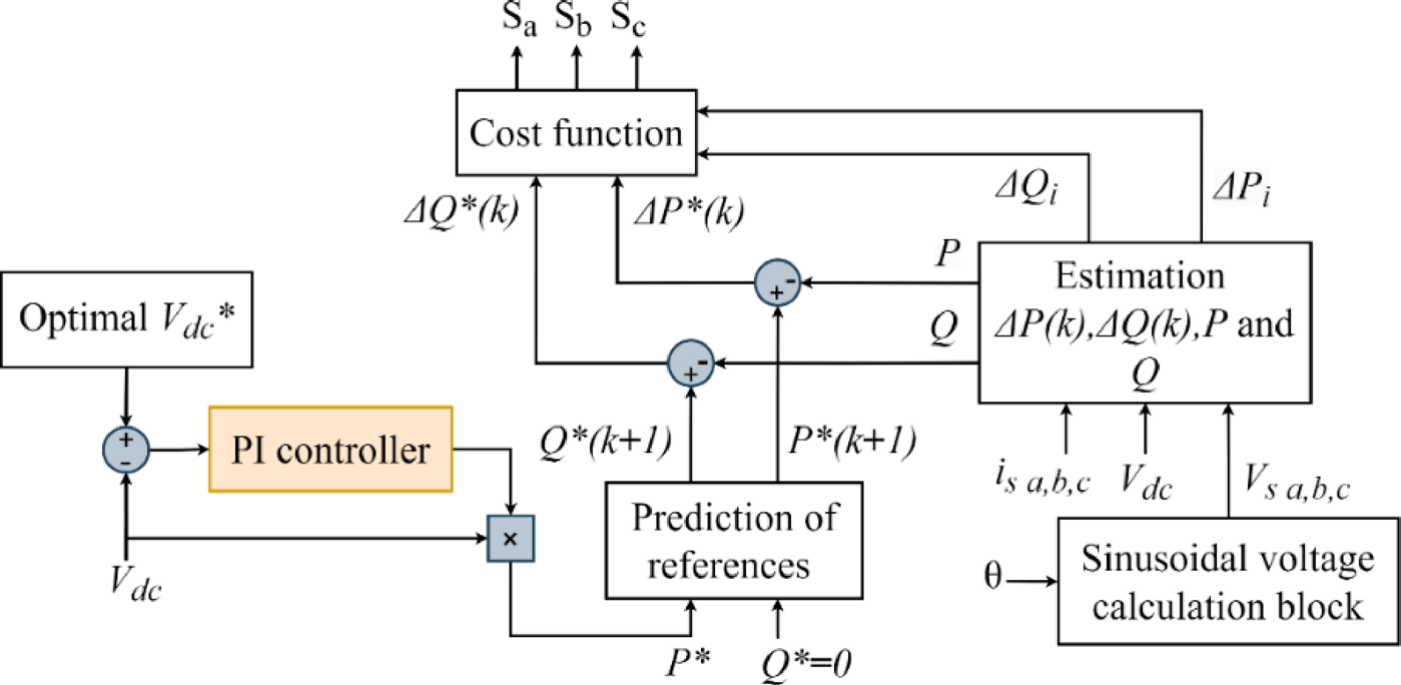
Overall structure of the PDPC control strategy.

Predictive Direct Power Control (PDPC) is considered one of the most effective control techniques for improving the performance of SAPF. The PDPC strategy relies on predicting changes in active power (P) and reactive power (Q) at each time instant. The overall structure of the PDPC-based control algorithm is illustrated in Fig. 2. This schematic diagram presents the interaction between the PI controller, the reference voltage optimization block, the power prediction module, and the cost function evaluation, highlighting the sequential flow of control signals within the SAPF system.

The cost function in this strategy compares the total of active and reactive powers with their predicted values to ascertain the ideal action that minimizes tracking errors and ensures the system’s optimal performance^12^, as seen in Eq. (3).

3 $${J_i}={(\Delta {P_i})^2}+{(\Delta {Q_i})^2}$$

Where, $\Delta {P_i}$ and $\Delta {Q_i}$represent the active and reactive power errors resulting from the application of vector$\overrightarrow {{v_i}}$. The optimal voltage vector is chosen as follows:

4 $${\vec {v}_{{\mathrm{opt}}}}=\arg {\hbox{min} _{{{\vec {v}}_i}}}{J_i},\;\;i=0,1,...,6$$

The proposed control strategy has been shown to be effective in enhancing system performance under a variety of conditions, as previously discussed in the literature. Nevertheless, its capacity to consistently improve power quality may be restricted by its dependence on a calculated *V*_dc_*, which may not account for real-time variations in system parameters. To address this challenge, implementing an optimization method to dynamically update the *V*_dc_* offers significant potential for reducing THD and ensuring more stable system performance.

### DC bus voltage regulation

**Fig. 3:**
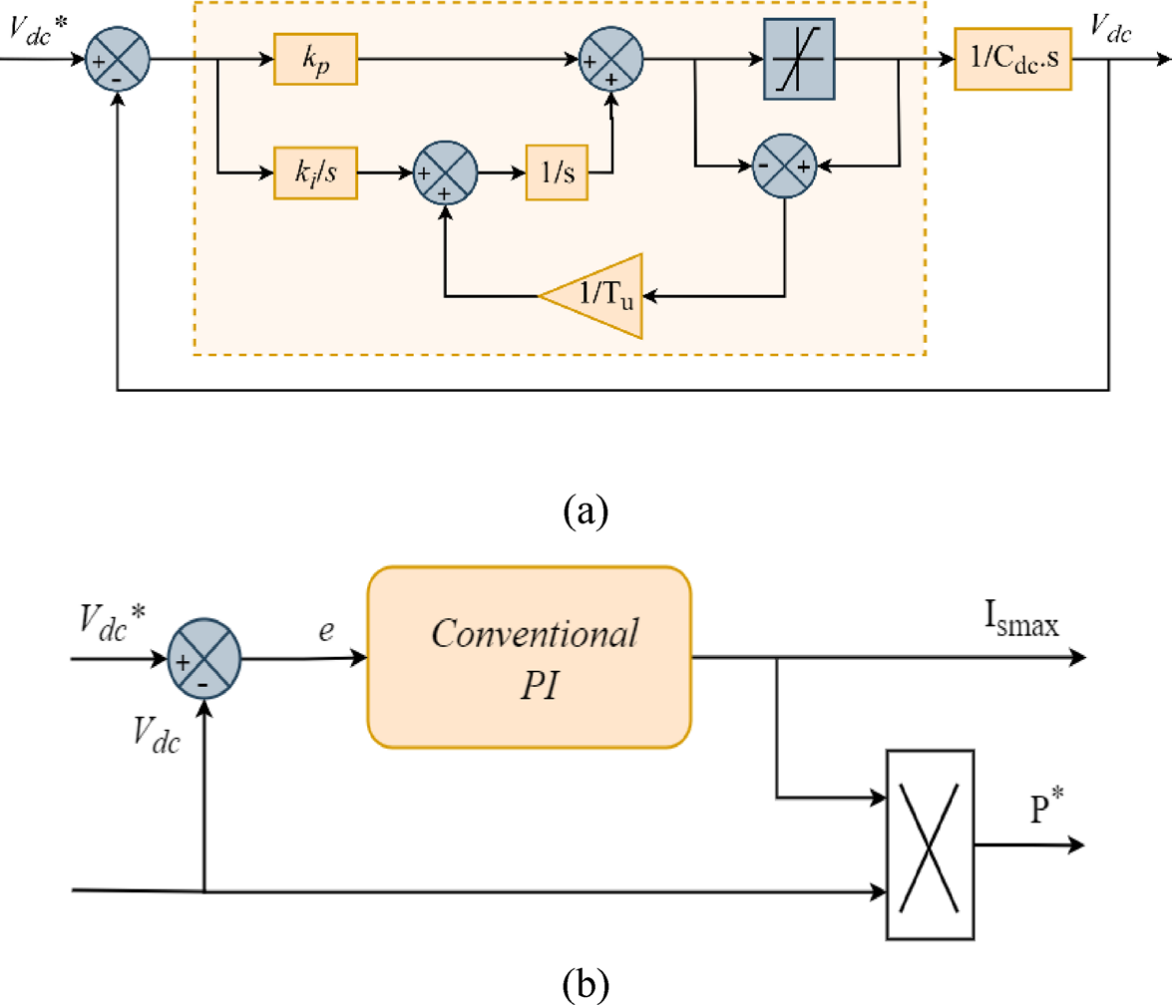
Block diagram: (**a**) PI Controller with Anti-windup Compensation for *V*_*dc*_ Regulation, (**b**) Outer Control Loop for *V*_*dc*_ using a PI Controller.

A PI controller with anti-windup correction keeps *V*_*dc*_ at a steady level. The anti-windup technique is based on a second integrator with a high loop gain (1/) to stop the output from becoming too high and make sure the voltage comes back quickly. The goal of this controller is to smooth out the changes and make sure that the PDPC method works at its best. To provide a clear insight into the implementation, Fig. 3a presents the detailed anti-windup PI structure used to regulate *V*_*dc*_. In addition, Fig. 3b shows how the PI controller generates the active power reference (P*), which is a key input in the PDPC control scheme for achieving effective compensation.

The transfer function of the PI controller is represented by Eq. (5). Where ${k_P}$denotes the proportional constant that promptly responds to discrepancies between the actual and reference voltages, while the integral constant ${k_i}$corrects for steady state errors^13^.

5 $${H_{pi}}\left( s \right)=\frac{{{V_{dc}}}}{{{V_{dc}}*}}=\frac{{\omega _{n}^{2}}}{{{s^2}+2 \cdot \xi \cdot {\omega _n} \cdot s+{\omega _n}}}$$

The values of ${k_P}$and ${k_i}$can be calculated using Eq. (6) and Eq. (7), respectively.

6 $${k_P}=2.\xi .{\omega _n}.{C_{dc}}$$

7 $${k_i}={C_{dc}}.{\omega _n}^{2}$$

Where, ${\omega _n}$ represents the natural frequency and $\xi$denotes the damping coefficient.

To support the operation of the filter, PV power is used to supply the filter with the necessary electrical power, reducing the need for energy consumption from the main grid.

### Modeling of PV system

**Fig. 4:**
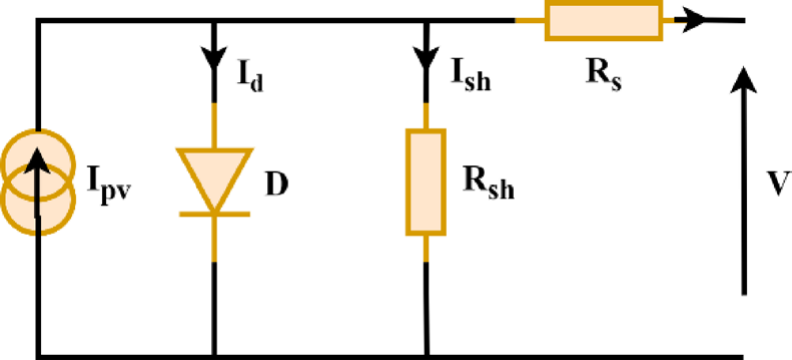
Single-diode equivalent circuit model of a PV Cell.

The PV cell is the fundamental unit in PV systems. It can be represented by several mathematical models, with the single-diode model, as shown in Fig. 4, being the most common. This model is represented by Eq. (8)^14^.

8 $${I_{pv}}={I_{ph}} - {I_{str}}\left( {{e^{\frac{{V+I{R_S}}}{{n{V_t}}}}} - 1} \right) - \frac{{V+I{R_S}}}{{{R_{sh}}}}$$

Solar cells are connected in series and parallel to form a photovoltaic array. Uniform solar irradiation of 600 W/m² is incident on the PV array, resulting in a power-voltage (P-V) curve with a single peak representing the Maximum Power Point (MPP). This study employed the Perturb and Observe (P&O) to optimize the performance of Maximum Power Point Tracking (MPPT)^15^, which facilitates the extraction of maximum available power^16^.

### Impact of PV power integration on DC-link voltage stability

The integration of PV systems with the grid provides a high energy level in the system, which raises several technical challenges affecting power quality. Among the most significant challenges is maintaining the stability of the Vdc under these dynamic conditions^17^. When the system is in a stable state without PV power injection, the DC-link power balance is:

9 $${P_{in,0}}={P_{out,0}}$$

Where, ${P_{in,0}}$represents the power input to the DC-link from the DC source, while ${P_{out,0}}$denotes the power drawn from the DC-link by the inverter.

The introduction of a PV source results in the injection of additional *P*_*pv*_ into the grid, leading to a power imbalance, as expressed in Eq. (10):

10 $${P_{in,new}}={P_{in,0}}+{P_{pv}}$$

If there is no immediate adjustment in the ${P_{out,0}}$, the *V*_*dc*_ increases due to excess energy accumulating in the DC-link capacitor (*C*_*dc*_). This can be expressed using the Eq. (11):

11 $$\frac{d}{{dt}}\left( {\frac{1}{2}{C_{dc}}V_{{dc}}^{2}} \right)={P_{in,new}} - {P_{out,new}}$$

Where, ${P_{out,new}}$represents the new power drawn from the DC-link by the inverter after the injection of *P*_*pv*_. If ${P_{out}}$is not adjusted, the accumulated energy causes a rise in *V*_*dc*_, resulting in a new ideal *V*_dc_*:

12 $$V_{{dc,new}}^{*}\left( {optimal} \right)>V_{{dc,0}}^{*}\left( {optimal} \right)$$

**Fig. 5:**
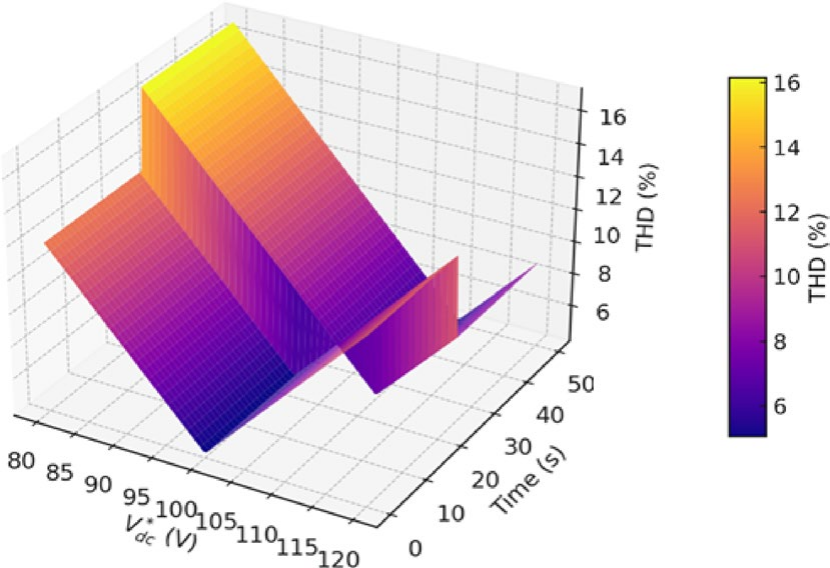
Heat map illustrating the impact of *V*_dc_* on THD before and after PV power injection.

This imbalance in power leads to an increase in the *V*_*dc*_ value, causing a rise in THD due to rapid voltage fluctuations. Figure 5 presents a heat map that helps illustrate the impact of *V*_dc_* on power quality in grid-connected PV systems. Before injecting PV power, an optimal value of *V*_dc_* is clearly observed, corresponding to the lowest THD. However, after PV power injection, this optimal *V*_dc_* shifts to a higher value than before. This change occurs due to variations in power flow within the system and its effect on the energy balance at the DC link.

The impact of the *V*_dc_* value on system quality highlights the need to determine this value accurately to maintain a minimum of harmonic distortions.

### Optimal DC bus reference voltage selection in PV systems

In this study, two conventional methods are applied to calculate the *V*_dc_* in a SAPF system when integrating PV power, and the impact of each method on system quality is analyzed. Subsequently, a new technique is proposed to determine the optimal *V*_dc_* with higher accuracy, and its effectiveness is evaluated by comparing it with conventional methods.

### Traditional methods for calculating the DC bus reference voltage

In previous studies, approximate methods based on mathematical equations have been used to calculate the value of *V*_dc_*. These equations rely on a set of fundamental system parameters, including the source voltage (*V*_*s*_). For example, the value of *V*_dc_* is calculated according to the first method using Eq. (13)^18^:

13 $${V_{dc}}*=\frac{{2\sqrt 2 }}{{1.155}} \cdot {V_{s(LL)}}$$

Additionally, there are other methods to determine the value of *V*_dc_* that require data extracted from simulations, such as the inverter output voltage (*V*_*f−max*_) value. In the second method, Eq. (14) illustrates how to calculate the value of *V*_dc_*, providing a different approach based on the system’s operational characteristics^16^.

14 $${V_{dc}}*=\sqrt 3 \cdot {V_{f\_\hbox{max} }}$$

Despite the effectiveness of these traditional methods, they lack the ability to adapt to dynamic changes in the grid, which are exemplified by the injection of PV power. This is due to the reliance of these traditional methods on fixed mathematical models for determining the *V*_dc_*, making them less efficient in handling sudden changes that the system may experience.

### *V*_dc_* optimization using mantis shrimp optimization algorithm (MShOA)

Given the PV energy injection into the SAPF system, and the resulting fluctuations that affect the stability of *V*_*dc*_, the Mantis Shrimp Optimization Algorithm (MShOA) is dynamically adapt to the *V*_dc_*. This metaheuristic algorithm is inspired by the complex visual system of this marine creature, which has exceptional light polarization analysis capabilities. The algorithm relies on three intelligent behaviors: foraging, attacking, and sheltering. Its primary goal is to minimize the THD in the grid current by instantly selecting the optimal value for the *V*_dc_*.

The following steps are used to implement the MAShA algorithm for *V*_dc_* optimization in real time:

A. **Population initialization**.

An initial population of solutions ${X_i}$is generated for =1,2,…,, within a defined search range bounded by $lb$ and $ub$:

15 $${X_i}=lb+rand \cdot (ub - lb)$$

Additionally, an initial Polarization Type Indicator (PTI) is assigned to each individual ${X_i}$:

16 $$PT{I_i}={\mathrm{round}}(1+2 \cdot rand)$$

B. **Eye angle and polarization type computation**.

To ascertain the polarization type detected by each eye, two polarization angles are computed, the left polarization angle (LPA) is calculated, which is determined by its similarity to the optimal solution, and the Right Polarization Angle (RPA), which is generated arbitrarily. The Left Angular Deviation (LAD) and Right Angular Deviation (RAD) are calculated by the algorithm from these values, which assess the degree to which each angle corresponds to predetermined polarization directions. The dominant eye is then identified for decision-making by comparing these deviations.

Then, the polarization types are assigned as follows^19^:

LPT:

17 $$LPT=\;\;\;{\kern 1pt} \left\{ {\begin{array}{*{20}{l}} {1,}&{{\mathrm{if}}\;\;\;{\kern 1pt} \frac{{3\pi }}{8} \leqslant LPA \leqslant \frac{{5\pi }}{8}}&{} \\ {2,}&{{\mathrm{if}}\;\;\;{\kern 1pt} 0 \leqslant LPA \leqslant \frac{\pi }{8}\;\;\;{\kern 1pt} {\mathrm{or}}\;\;\;{\kern 1pt} \frac{{7\pi }}{8} \leqslant LPA \leqslant \pi }&{} \\ {3,}&{{\mathrm{if}}\;\;\;{\kern 1pt} \frac{\pi }{8}<LPA<\frac{{3\pi }}{8}\;\;\;{\kern 1pt} {\mathrm{or}}\;\;\;{\kern 1pt} \frac{{5\pi }}{8}<LPA<\frac{{7\pi }}{8}}&{} \end{array}} \right.$$

RPT:

18 $$RPT=\;\;\;{\kern 1pt} \left\{ {\begin{array}{*{20}{l}} {1,}&{{\mathrm{if}}\;\;\;{\kern 1pt} \frac{{3\pi }}{8} \leqslant RPA \leqslant \frac{{5\pi }}{8}}&{} \\ {2,}&{{\mathrm{if}}\;\;\;{\kern 1pt} 0 \leqslant RPA \leqslant \frac{\pi }{8}\;\;\;{\kern 1pt} {\mathrm{or}}\;\;\;{\kern 1pt} \frac{{7\pi }}{8} \leqslant RPA \leqslant \pi }&{} \\ {3,}&{{\mathrm{if}}\;\;\;{\kern 1pt} \frac{\pi }{8}<RPA<\frac{{3\pi }}{8}\;\;\;{\kern 1pt} {\mathrm{or}}\;\;\;{\kern 1pt} \frac{{5\pi }}{8}<RPA<\frac{{7\pi }}{8}}&{} \end{array}} \right.$$

Ultimately, the effective polarization type (PTI) is determined by the lesser angular deviation:

19 $$PT{I_i}=\left\{ {\begin{array}{*{20}{l}} {LP{T_i}}&{{\mathrm{if}}LA{D_i}<RA{D_i}}&{} \\ {RP{T_i}}&{{\mathrm{if}}LA{D_i}>RA{D_i}}&{} \end{array}} \right.$$

C. **Position update based on behavior**.

According to the value of PTI, each individual’s position is updated using one of the following behavioral rules:

If PTI = 1 (Foraging behavior):

20 $$\begin{gathered} X_{i}^{{t+1}}={X_{best}} - v \cdot {\mathrm{rand}} \cdot ({X_r} - X_{i}^{t}),\;\;\;{\kern 1pt} rand \in [ - 1,1] \hfill \\ v=X_{i}^{t} - {X_{best}} \hfill \\ \end{gathered}$$

If PTI = 2 (Attacking behavior):

21 $$X_{i}^{{t+1}}={X_{best}} \cdot \cos \theta$$

If PTI = 3 (Sheltering behavior):

22 $$\begin{gathered} X_{i}^{{t+1}}={x_{best}}+rand \cdot ({x_{best}}),\;\;\;{\kern 1pt} rand \in [0,0.3] \hfill \\ X_{i}^{{t+1}}={x_{best}} - rand \cdot ({x_{best}}),\;\;\;{\kern 1pt} rand \in [0,0.3] \hfill \\ \end{gathered}$$

Where:

$X_{i}^{t}$ The current position of the ${i^{th}}$ ith individual at iteration *t*,

$X_{i}^{{t+1}}$ The updated position,

${x_{best}}$ The best solution,

${X_r}$ A randomly selected individual from the population,

$rand$ A uniformly distributed random number.

This mechanism enables the algorithm to adaptively explore and exploit the search space in accordance with the behavior dictated by PTI.

D. **Evaluation and best solution selection**.

The objective function value (THD level) for all individuals is assessed during each iteration. The optimal candidate solution is revised in accordance with:

23 $${X_{{\mathrm{best}}}}=\arg \hbox{min} \cdot f({X_i})$$

**Fig. 6:**
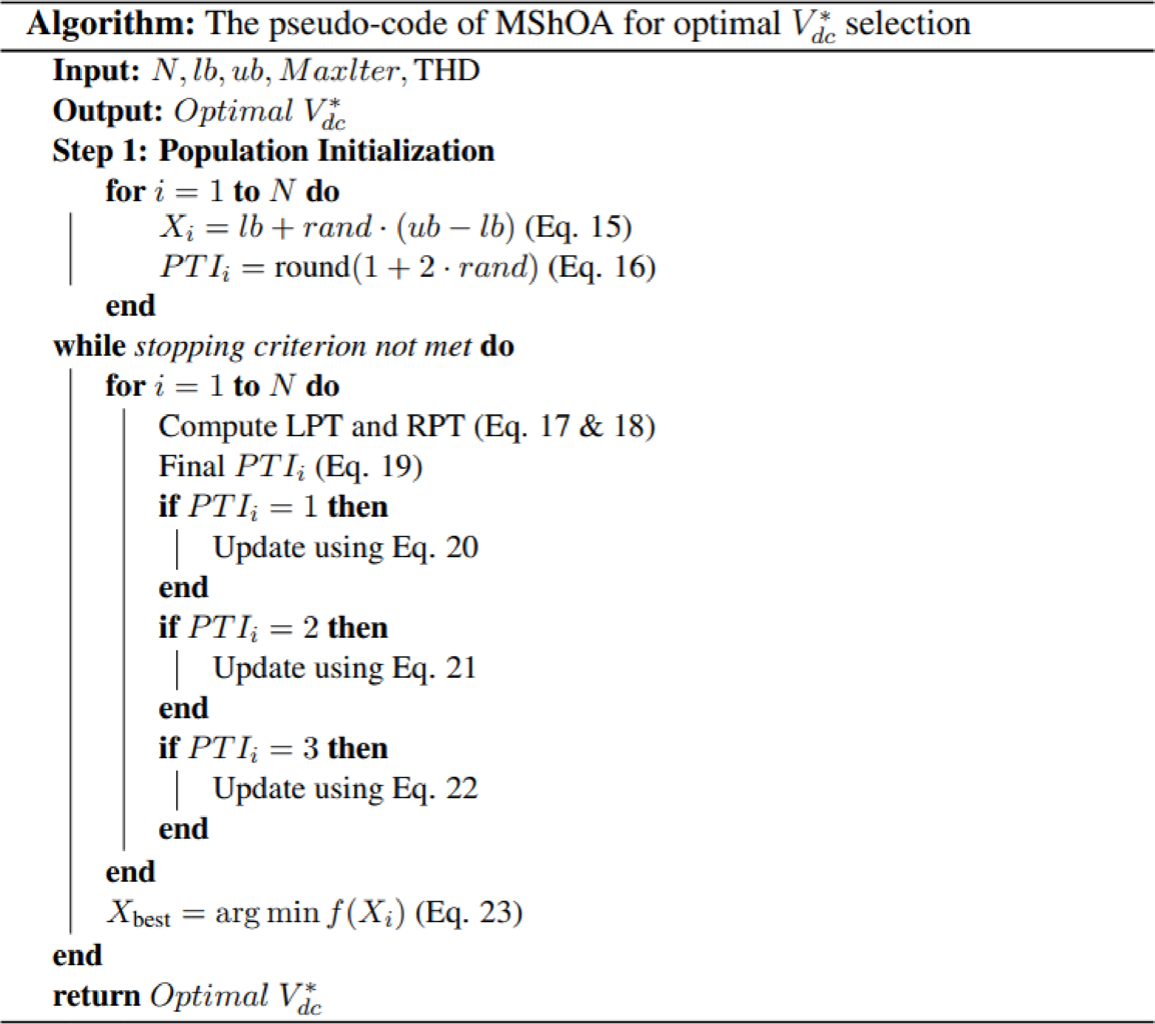
Pseudo-code of the MShOA for Optimal *V*_dc_* Determination in SAPF.

This process repeats until a maximum number of iterations is reached or a minimum THD value is achieved. Finally, the optimal solution ${X_{{\mathrm{best}}}}$is adopted as the final *V*_dc_* used in the SAPF system. To provide a clearer view of the implementation procedure, the pseudo-code corresponding to the proposed MShOA-based is presented in Fig. 6.

### Simulation results

This section presents a series of simulation results to study the effect of the *V*_dc_* value in the SAPF system under the influence of PV energy injection. To accurately capture the harmonic dynamics and switching behavior of the system, a detailed switching model of the three-phase VSI and SAPF was used, incorporating Pulse Width Modulation (PWM) and nonlinear load modeling. The simulation is designed to reflect realistic operating conditions, and all simulations were performed in the MATLAB/Simulink environment using the system parameters shown in Table 1.

**Table 1:** System’s parameters.

Parameters		Values
Grid Side		
Source voltage Vs		25 *V*
Source resistance *R*_*S*_		0.1 *Ω*
Source inductance *L*_*s*_		0. 1 *mH*
Supply frequency *f*		50 *Hz*
DC Bus reference voltage (Approach 1) *V*_dc_^*^_APP1_		100 *V*
DC Bus reference voltage (Approach 2) *V*_dc_^*^_APP2_		106 *V*
DC Bus reference voltage (IGWO) *V*_dc_^*^_MShOA_ (Before injecting PV power)		113.5 *V*
DC Bus reference voltage (IGWO) *V*_dc_^*^_MShOA_ (After injecting PV power)		147.8 *V*
DC Bus capacitor *C*_*dc*_		1100 µ*F*
Filter inductance *L*_*f*_		8.5 *mH*
Filter resistance *R*_*f*_		0.1 *Ω*
Proportional gain *k*_*p*_		0.1521
Integral gain *k*_*i*_		2.1713
Sampling time *T*_*s*_		4.5 µs
Load inductance *L*_*l*_		8.5 *mH*
Load resistance *R*_*l*_		5 *Ω*
PV Side		
Open circuit voltage V_oc_		36.6 V
Court circuit current I_cc_		7.97 A
Current at maximum power point I_mpp_		7.27 A
Voltage at maximum power point V_mpp_		29.3 V

### Traditional methods analysis

In this analysis, the efficacy of two conventional approaches utilized to determine *V*_dc_* in the SAPF system is assessed. It is also investigated how the *V*_dc_* value affects the grid current’s THD. Additionally, these two conventional approaches will be evaluated in the context of PV power injection.

**Fig. 7:**
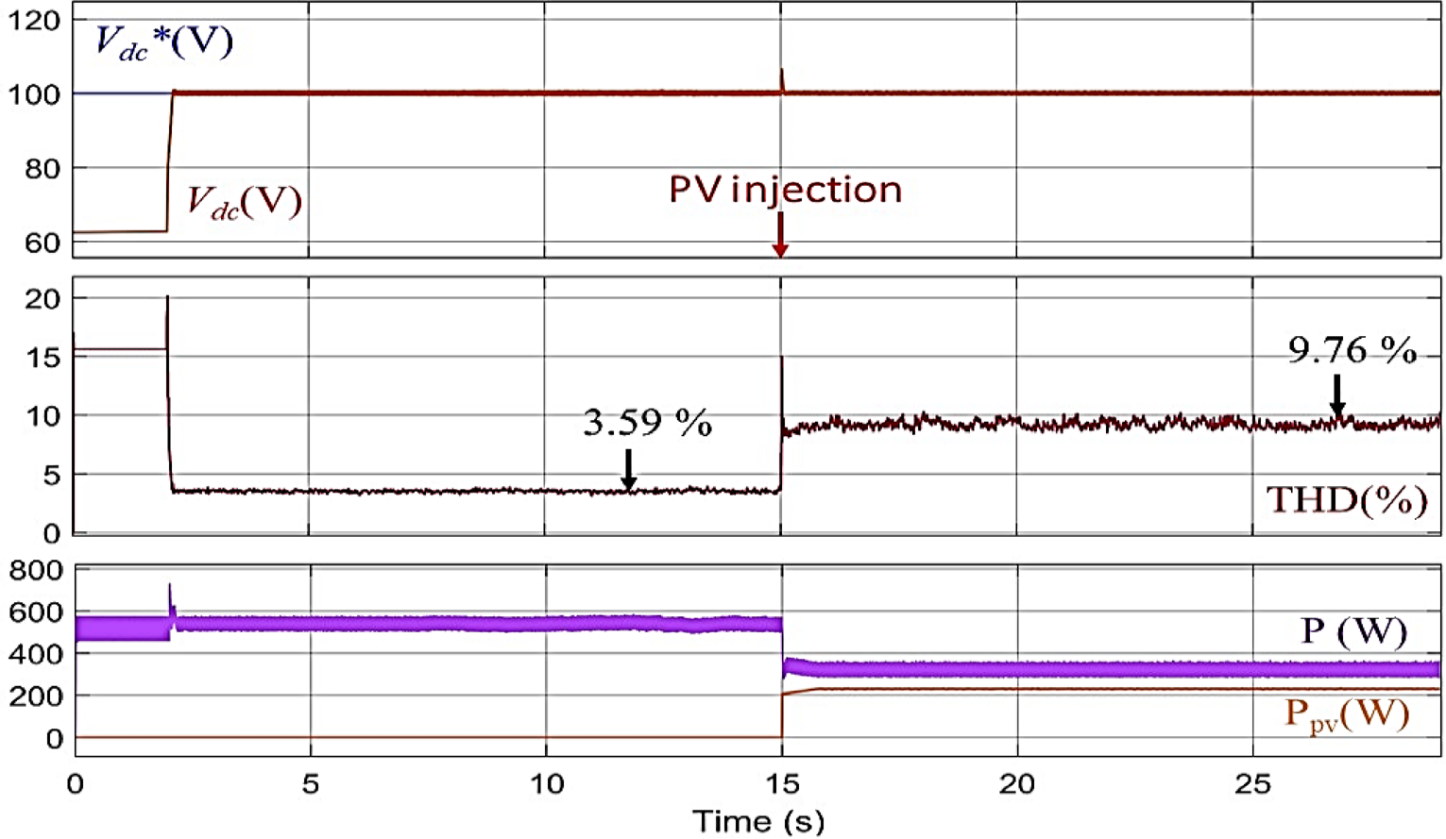
Performance of the SAPF using the first conventional method before and after PV power injection.

**Fig. 8:**
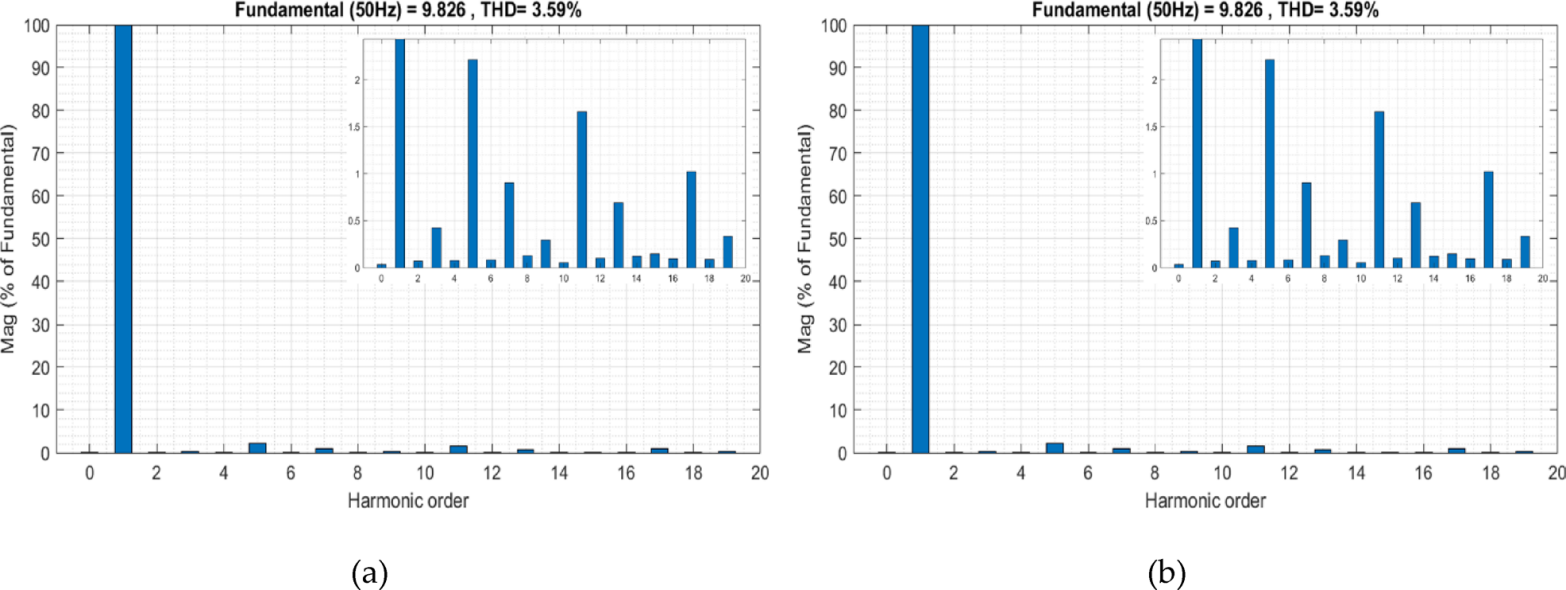
Grid current THD evolution using the first conventional method: (**a**) Before PV injection, (**b**) After PV injection.

The first method used Eq. (13) to obtain the correct value of Vdc*. The result was a *V*_dc_* value of 100 volts, As illustrated in Fig. 7. Before activating the active filter, the distortion level in the grid is high due to the absence of any effective correction for these distortions caused by nonlinear loads. THD of the current was measured at 16%. After activating the filter at T = 2 s, the *V*_*dc*_ closely follows its reference value *V*_dc_* during the initial phase, maintaining a relatively stable level. Additionally, THD is effectively reduced to 3.59%, as shown in Fig. 8a. Indicating the efficiency of the PDPC technique. However, since the value of *V*_dc_* was calculated using the first approach, the system’s performance remains constrained by the accuracy of this method and its effectiveness under dynamic operating conditions or when external disturbances are applied to the system. at T = 15 s, a noticeable fluctuation in the *V*_*dc*_ curve is observed, indicating the system’s struggle to maintain stability under the dynamic conditions introduced by PV power injection. In parallel, the THD rises again to 9.76%, as illustrated in Fig. 8b., revealing a significant deterioration in power quality.

**Fig. 9:**
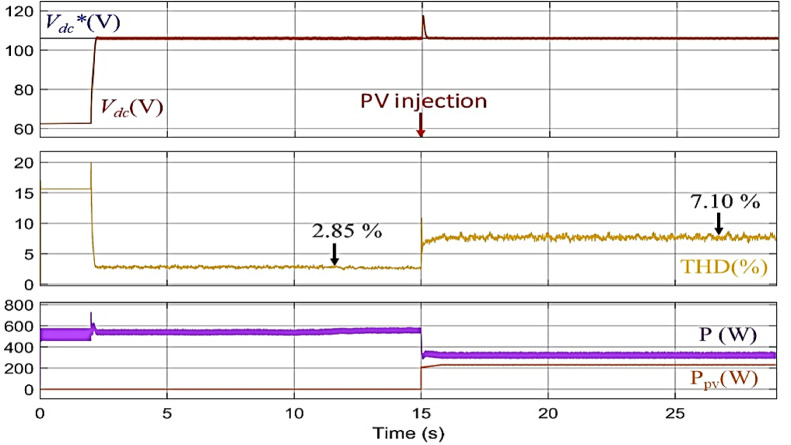
Performance of the SAPF using the second conventional Method before and after PV power injection.

**Fig. 10:**
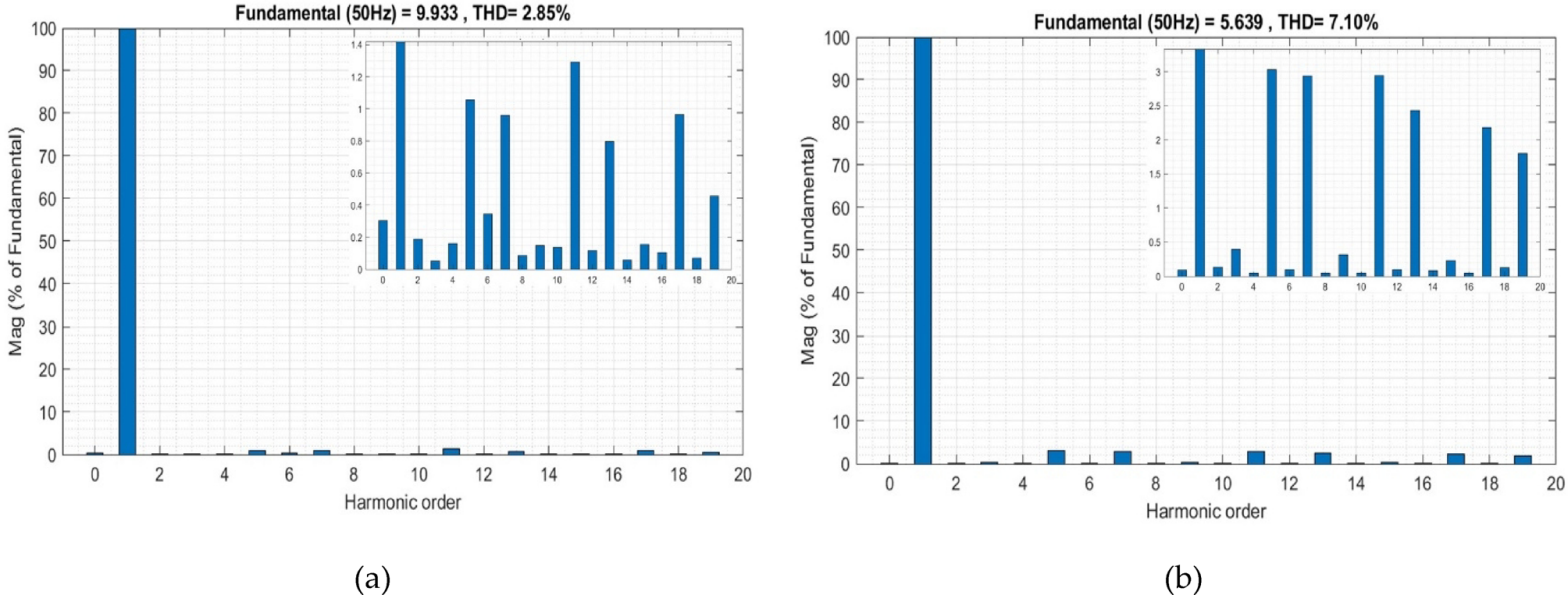
Grid current THD evolution using the second conventional method: (**a**) Before PV injection, (**b**) After PV injection.

By transitioning to the second conventional method while maintaining the same previous test conditions, the impact of the *V*_dc_* value on the system is assessed. Initially, the *V*_dc_* value is calculated using Eq. (13), resulting in a reference value of 106 V. As shown in Fig. 9, after activating the filter at 2 s, an improvement in the stability of the *V*_dc_* observed, leading to a reduction in the THD to 2.85%, as illustrated in Fig. 10a. However, when PV power is injected at T = 15 s, similar challenges arise. The *V*_*dc*_ is affected by sudden changes, and the THD increases to 7.10%, as seen in Fig. 10b. Indicating the impact of dynamic conditions on the efficiency of the second approach. second.

The simulation results for both the first and second approaches for calculating the value of *V*_dc_* reveal that their effectiveness is limited in accurately determining this value, especially under dynamic conditions or external disturbances affecting the system. This is particularly evident during PV power injection, where the THD increased significantly in both methods. In light of these findings, it becomes evident that more flexible and intelligent techniques are required, ones that can perform real-time optimization and account for power quality indicators.

### Proposed technique analysis

To validate the effectiveness of the proposed optimization-based control strategy, this section presents a detailed analysis of the applied algorithms used to determine the optimal reference voltage *V*_dc_*. Two intelligent optimization methods were considered: the MShOA, which forms the basis of the proposed approach, and the Whale Optimization Algorithm (WOA), implemented for comparison and verification purposes. Each algorithm is analyzed separately under identical operating conditions to evaluate its capability in minimizing THD and maintaining *V*_*dc*_ stability.

#### Performance of the proposed MShOA-based method

**Fig. 11:**
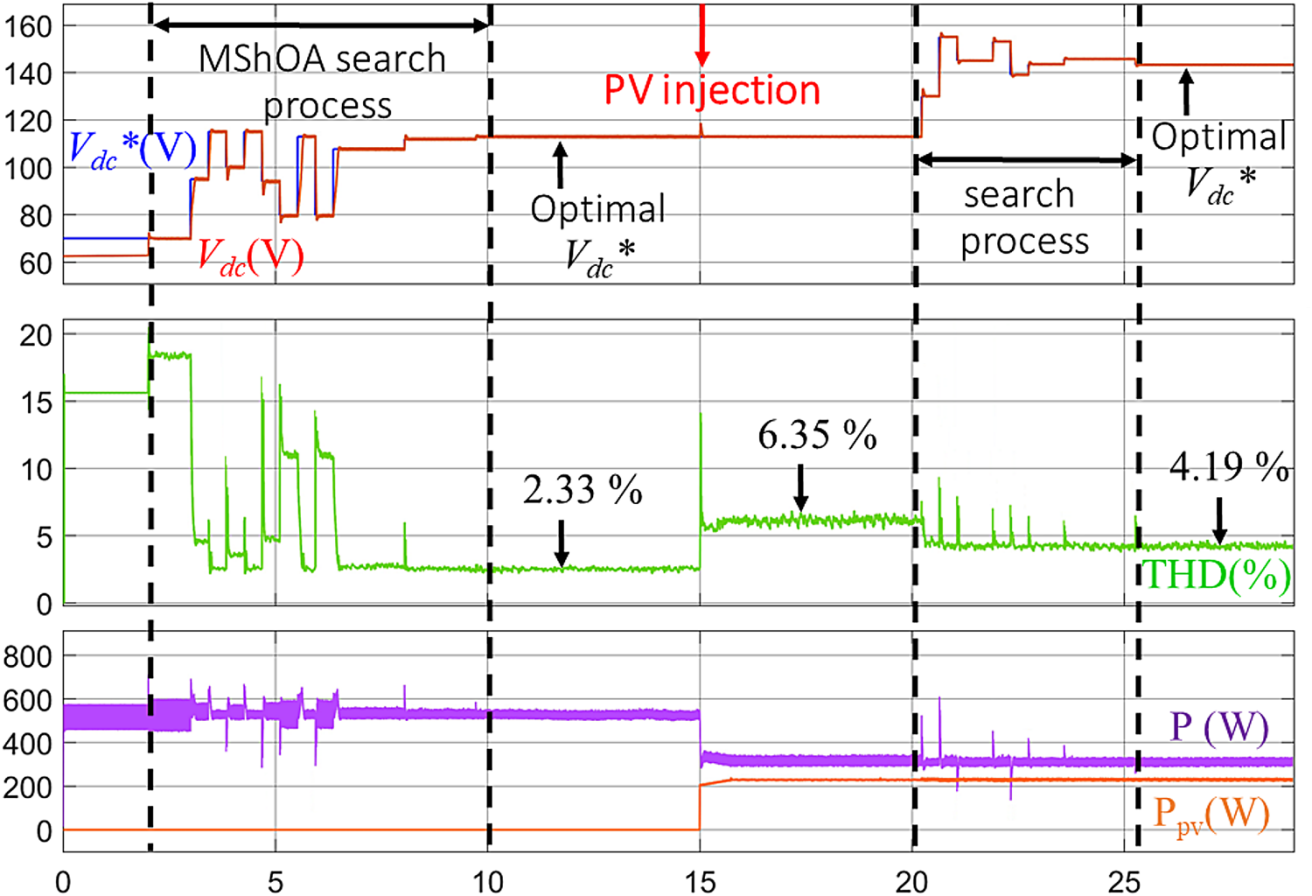
Real-Time evolution of *V*_dc_* and *V*_*dc*_ with the proposed MShOA under PV injection conditions.

To overcome the limitations of traditional methods, the proposed technique, which relies on the MShOA, was applied to dynamically determine the optimal *V*_dc_* in real time. To ensure a fair comparison, the simulation was conducted under the same test conditions as those used for the first and second conventional approaches, including PV power injection at T = 15 s As shown in Fig. 11, the evolution of both *V*_*dc*_ and *V*_dc_* over time is presented, along with their impact on the THD.

**Fig. 12:**
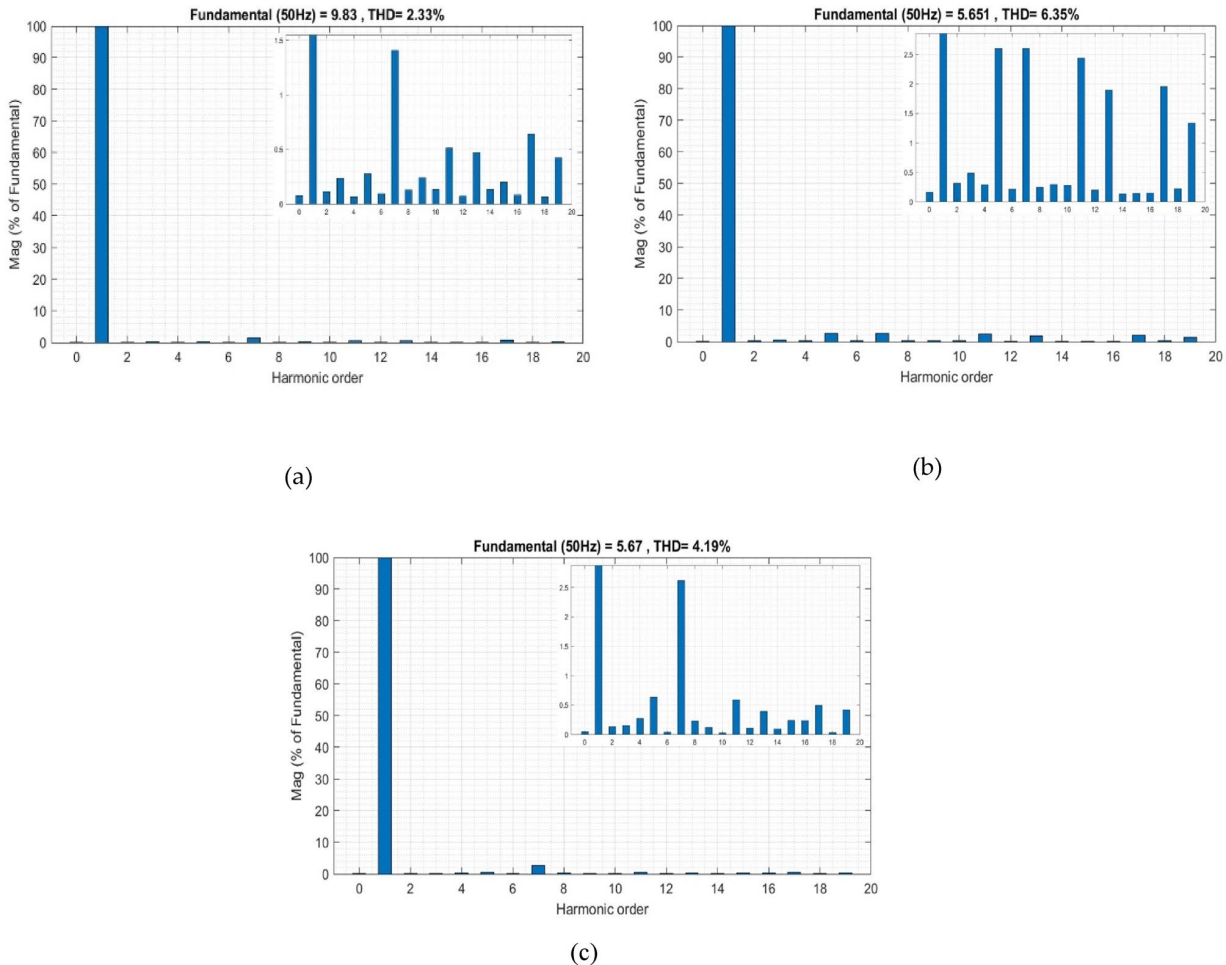
THD improvement using MShOA: (**a**) Before PV injection, (**b**) After PV injection, (**c**) Post re-optimization.

Before activating the active filter, the THD was high, and the *V*_dc_* was unstable at its reference value *V*_dc_*. After activating the filter at T = 2 s, the MShOA initiated the search process for the optimal *V*_dc_*. At the beginning of this process, an increase in THD is observed, which corresponds to unsuitable *V*_dc_* values for the system. As the MShOA continued its search, the value of *V*_dc_* was gradually adjusted, positively impacting the THD by reducing it to 2.33%, as shown in Fig. 12a. This is achieved by relying on the objective function of the MShOA, which guides the search process to select the *V*_dc_* value that corresponds to the lowest THD.

In the second phase, PV power is injected at T = 15 s. The MShOA is programmed to restart the search process at T = 20 s to highlight the difference in THD levels before and after applying the algorithm under changing conditions. Initially, after PV injection, a significant increase in THD to 6.35% is observed, as shown in Fig. 12b. This rise in THD is attributed to the fact that the previously determined *V*_dc_* value is no longer suitable for the system under these new conditions.

Subsequently, the MShOA begins searching for the appropriate *V*_dc_* value that matches the new conditions. Initially, fluctuations in THD values appear, as the search process continues, the *V*_dc_* values gradually adapt to the new operating conditions, leading to a reduction in THD to 4.19%, as illustrated in Fig. 12c. This confirms the effectiveness of the proposed technique in determining the optimal *V*_dc_* value and its ability to dynamically adapt to real-time operating changes, thereby improving power quality efficiently under these varying conditions.

#### Implementation of the Whale optimization algorithm (WOA)

To expand the scope of the analysis, the WOA was applied to determine the optimal *V*_dc_*. The WOA mimics the bubble-net hunting behavior of humpback whales and alternates between exploration and exploitation phases to locate the optimal solution within a defined search space. In this study, the same system parameters, operating conditions, and objective function used with the MShOA were maintained to ensure consistency in evaluation.

**Fig. 13:**
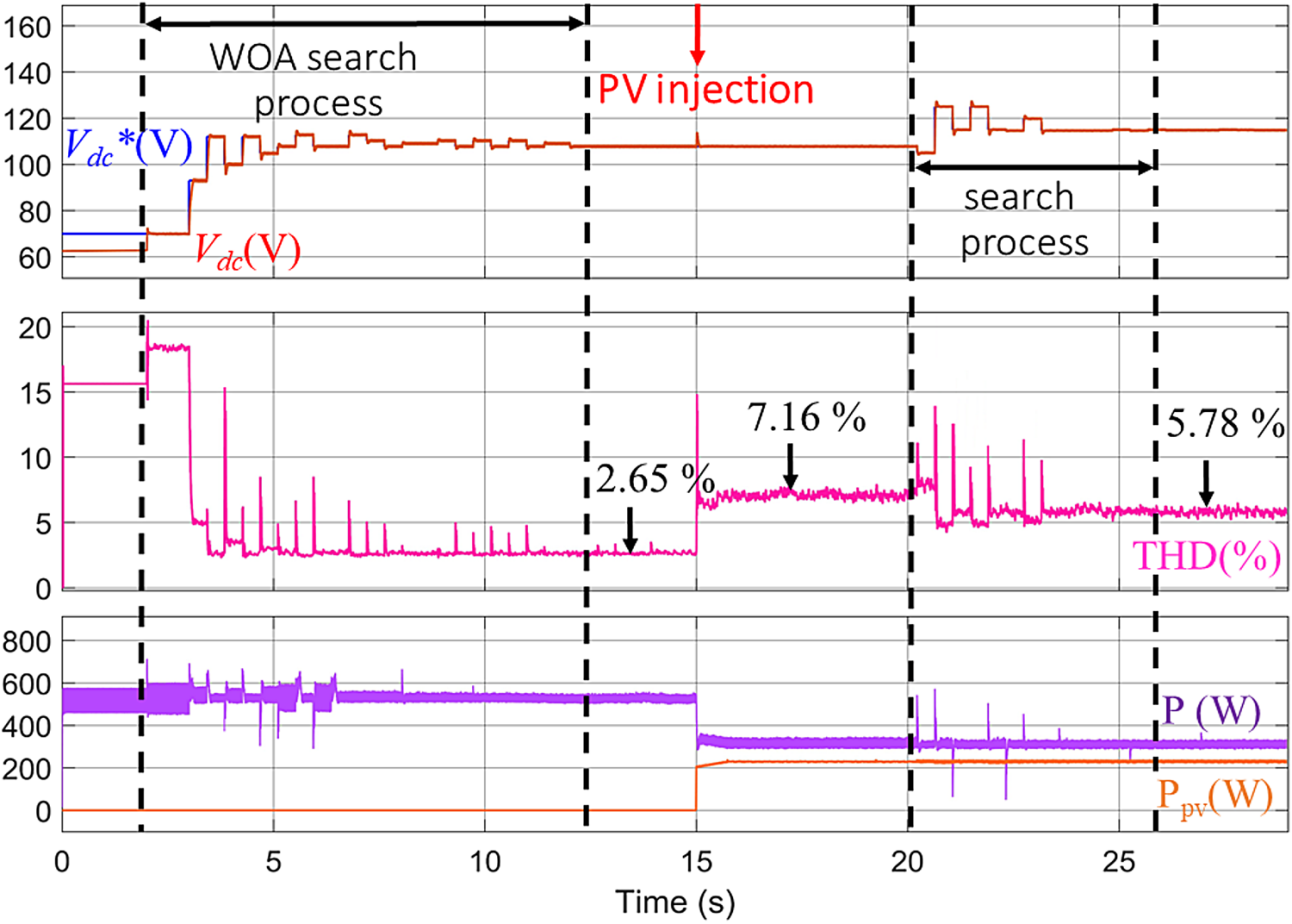
Real-Time evolution of *V*_dc_* and *V*_*dc*_ with the WOA under PV injection conditions.

**Fig. 14:**
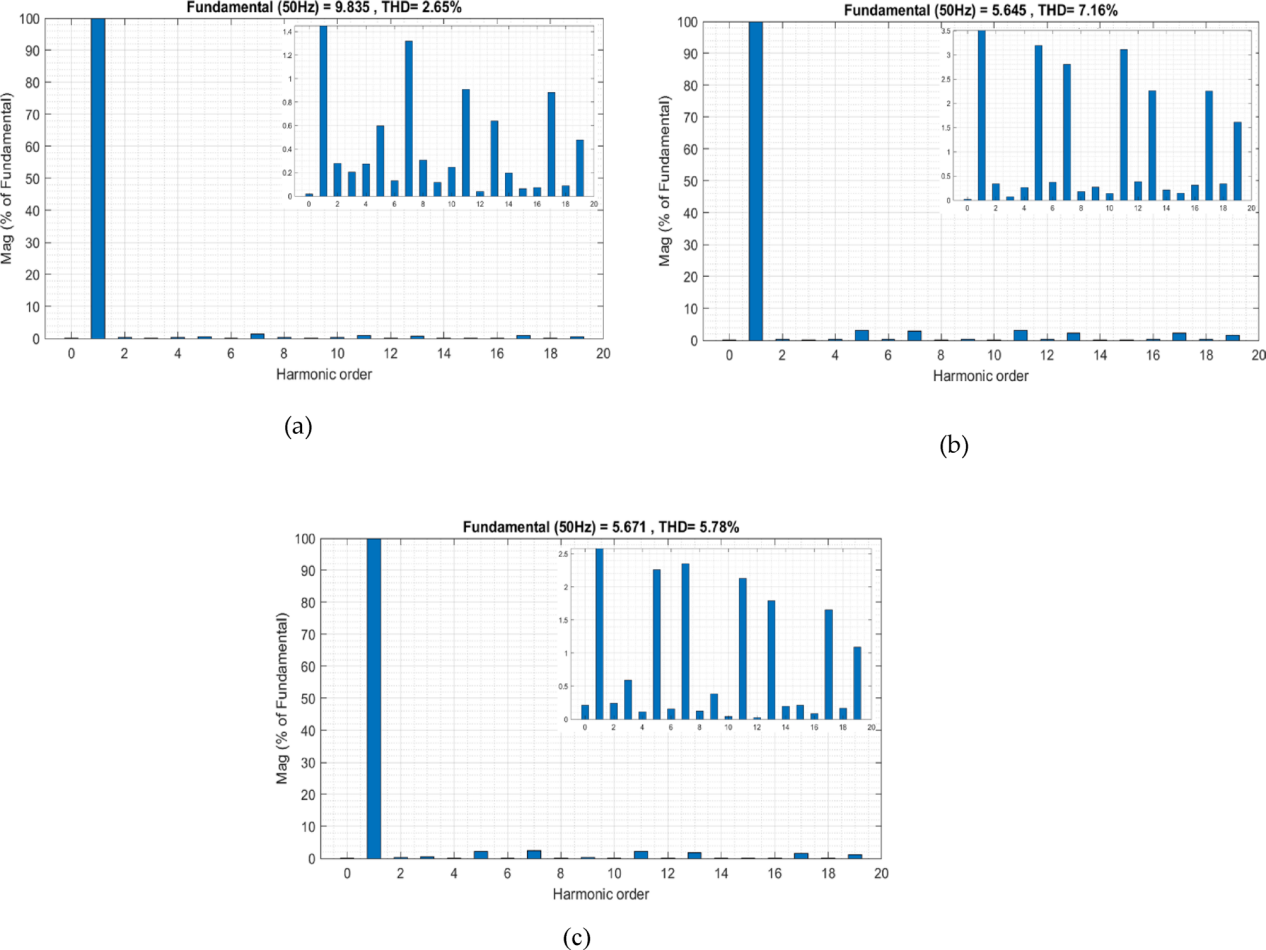
THD improvement using WOA: (**a**) Before PV injection, (**b**) After PV injection, (**c**) Post re-optimization.

Simulation results obtained using the WOA demonstrate its ability to converge toward a feasible *V*_dc_* value and effectively reduce the THD of the source current, as illustrated in Fig. 13. Before activating the active filter, the THD level was high; once the filter was activated at T = 2 s, the WOA began its optimization process, reducing the THD to 2.65%, as shown in Fig. 14a. In the second phase, when PV power was injected at T = 15 s, a noticeable increase in THD to 7.16% was observed, as illustrated in Fig. 14b. This increase occurs because the previously determined *V*_dc_* no longer matches the system’s new operating conditions. As the WOA continues its iterative search, *V*_dc_* adapts to the new conditions, resulting in a reduction of THD to 5.78%, as shown in Fig. 14c.

These results confirm the capability of the WOA to dynamically adjust *V*_dc_* and maintain acceptable harmonic distortion levels, although its convergence speed and final performance remain lower than those achieved with the proposed MShOA-based method.

### Comparative evaluation

To validate the effectiveness of the proposed MShOA-based technique in determining the optimal value of *V*_dc_*, a comparative analysis was conducted with another optimization-based approach using the WOA. The inclusion of the WOA method serves to further verify the superiority of the MShOA strategy under identical conditions. Additionally, two conventional methods were analyzed for reference and benchmarking purposes.

**Fig. 15:**
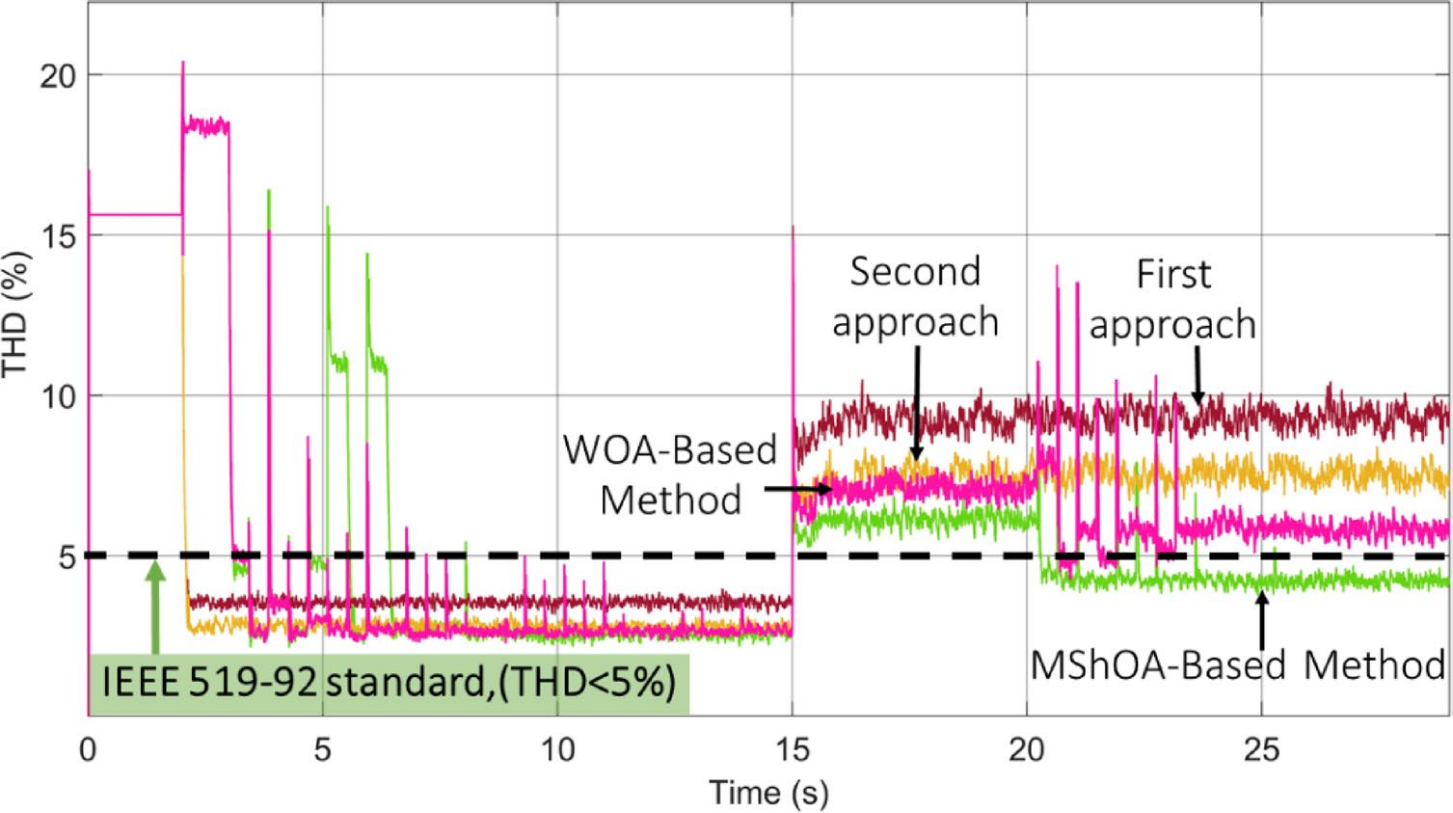
Comparative Analysis of THD Reduction.

As illustrated in Fig. 15, the proposed MShOA-based technique demonstrates a distinctly superior performance compared to all other approaches. When the filter is activated at T = 2 s, the MShOA achieves the lowest THD value of 2.33%, followed by the WOA-based method at 2.65%, while the conventional techniques record significantly higher distortion levels of 3.59% and 2.85%, respectively. This initial result clearly indicates the higher precision and faster convergence of the MShOA in identifying the optimal reference voltage. During PV power injection at T = 15 s, the advantage of the MShOA becomes even more evident. While both optimization algorithms adapt to dynamic variations, the WOA-based method reduces THD to 5.78%, whereas the proposed MShOA achieves a superior reduction to 4.19%, maintaining distortion within the acceptable limit defined by the IEEE 519 − 92 standard (< 5%).

To provide a clearer quantitative comparison, Table 2 summarizes the THD values obtained for all evaluated methods, both before and after PV power injection.

**Table 2:** THD performance comparison before and after PV injection using conventional methods, WOA, and MShOA.

Method	THD before PV injection (%)	THD after PV injection (%)
First traditional approach	3.59	9.76
Second traditional approach	2.85	7.10
WOA-based method	2.65	5.78
Proposed MShOA-based method	2.33	4.19

These outcomes confirm that, although the WOA improves upon conventional approaches, the MShOA-based strategy consistently provides the best dynamic adaptability, faster optimization response, and lowest harmonic distortion, proving its higher robustness and effectiveness in real-time voltage control and power quality enhancement.

**Fig. 16:**
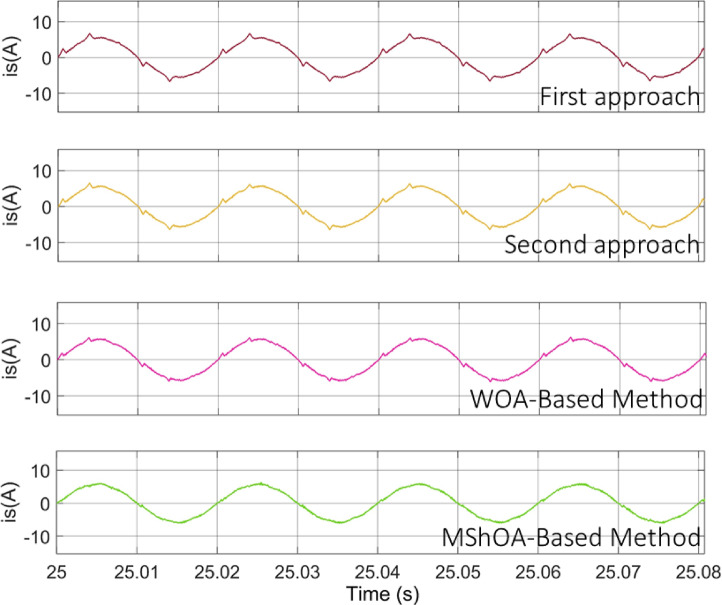
Improvement of source current waveform using the proposed MShOA Technique under PV injection.

Figure 16. presents a comparison of the ${i_s}\left( t \right)$ waveform after PV power injection into the system using two conventional methods, as well as two optimization-based techniques. The WOA-based approach and the proposed MShOA-based method. It is evident that the conventional methods fail to effectively correct the current waveform, as noticeable distortions remain, negatively impacting the overall power quality.

In contrast, the optimization-based approaches provide a much smoother and more sinusoidal waveform. Among them, the proposed MShOA-based technique delivers the best performance, producing a source current waveform that is the closest to the ideal sinusoidal form. This confirms its superior ability to minimize harmonic distortion and maintain stable operation under dynamic conditions, surpassing both the WOA and conventional approaches in terms of accuracy and efficiency.

## Conclusion

This work presented a novel real-time optimization strategy for dynamically adjusting the DC bus reference voltage (*V*_dc_*) in a three-phase Shunt Active Power Filter (SAPF) under photovoltaic (PV) power injection conditions. The proposed technique is based on the Mantis Shrimp Optimization Algorithm (MShOA).

The simulation results confirmed the limitations of existing approaches in adapting *V*_dc_* under dynamic operating conditions. Both conventional analytical methods and the Whale Optimization Algorithm (WOA) achieved acceptable performance only under steady state, but they failed to maintain power quality when sudden PV power injections introduced disturbances, leading to noticeable increases in Total Harmonic Distortion (THD) and voltage instability. In contrast, the proposed MShOA-based strategy demonstrated a superior ability to accurately and adaptively adjust Vdc* in real time, achieving lower THD values, enhanced system stability, and full compliance with IEEE 519 − 92 harmonic standards under PV injection.

Future research will explore the integration of experimental validations and hardware implementation of the proposed approach, as well as extending the optimization framework to multi-objective scenarios involving power factor correction and energy efficiency.

## Data Availability

Correspondence and requests for materials should be addressed to Afghoul Hamza.
